# Ultrasonic-assisted acidified aqueous two-phase extraction for one-step production and recovery of flavonoid aglycones from *Malvaviscus arboreus* Cav. flower: Process exploration, composition analysis and activity validation

**DOI:** 10.1016/j.ultsonch.2025.107545

**Published:** 2025-08-31

**Authors:** Tiefeng Yuan, Chen Lin, Qingqing Yu, Xingyu Shi, Peiyi Jin, Jilong Huang, Liping Wang, Huajun Fan

**Affiliations:** a Guangdong Provincial Key Laboratory of Chemical Measurement and Emergency Test Technology, Institute of Analysis, Guangdong Academy of Science (China National Analytical Center), Guangzhou 510070, China; b School of Pharmacy, Guangdong Pharmaceutical University, Guangzhou 510006, China

**Keywords:** *Malvaviscus arboreus *Cav. flower, Ultrasonic-assisted acidified aqueous two-phase extraction, Flavonoid aglycones, Process mechanism, Enzyme inhibitory activity, Molecular docking analysis

## Abstract

Using a polyethylene glycol (PEG)/ammonium sulfate system, a novel approach for one-step extraction and separation of the flavonoid aglycones from *Malvaviscus arboreus* Cav. flower (MACF) was developed by combining ultrasonic-assisted extraction (UAE) with acidified aqueous two-phase extraction (AATPE). Under the optimized UAE-AATPE conditions of PEG 600 of 22.8 % (w/w), ammonium sulfate of 22.5 % (w/w), 1.5 mol/L HCl, hydrolysis temperature of 85 ℃, and reaction time of 60 min, MACF flavonoid glycosides were not only fully extracted but also completely hydrolyzed, and the produced aglycones were purified by biphasic separation with a yield of 35.90 ± 0.96 mg/g. The mechanism in the UAE-AATPE process including UAE and ultrasonic-assisted acid hydrolysis (UAAH), was explored by investigating the different procedures and chemical composition under an ultrasonic field. The deglycosylated/glycosylated flavonoids were identified and quantified by high-resolution ultrahigh-performance liquid chromatography with quadrupole orbitrap mass spectrometry (UHPLC-Q/Orbitrap-MS) and UHPLC-DAD, revealing that MACF flavonoids were mainly composed of pelargonidin, kaempferol, catechin, cyanidin and quercetin in descending order of their content. The inhibitory assay on pancreatic lipase (PL) confirmed that the activity of the extracted aglycones with IC_50_ of 563.01 ± 280.63 μg/mL was stronger than their glycosides. Kinetic and molecular docking analyses further explored and validated the PL inhibitory mechanism. The above results proved that UAE-AATPE integrating the multiple procedures, including extraction, hydrolysis and separation, greatly simplified the complex procedures and significantly improved extraction efficiency, providing a simple, rapid and efficient alternative for direct obtainment of the flavonoid aglycones from MACF.

## Graphical abstract

**Figure:**
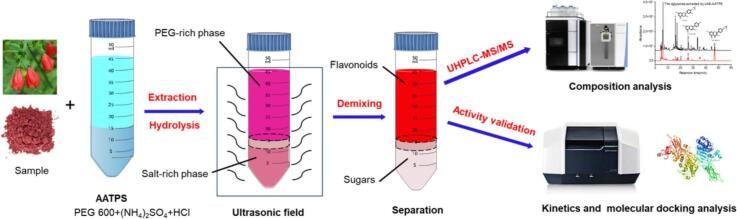

## 1 Introduction

Flowers have been widely used for decorative and culinary purposes since ancient times, and long before the chemical composition was identified, they were used as enhancers of color and aroma or to improve the appearance of food [1]. From the chemical perspective, flowers can be considered as a health promoter, which is attributed to the abundant array of bioactive components, such as flavonoids and polysaccharides [2,3]. *Malvaviscus arboreus* Cav. flower (MACF), an edible flower widely cultivated in Brazil and southern China, is used for jelly, salad, tea, syrup, light sauce, and as a natural red food colorant. Its unique flavor lends itself perfectly to teas, salads, and curries, where it adds a delicate sweetness and floral aroma, enhances the freshness of the greens, and provides a subtly sweet balance to the flavors [[4], [5], [6], [7]]. Furthermore, previous studies revealed the presence of a series of polyphenols derivatives, especially flavonoids and anthocyanins, and the flavonoid extracts derived from MACF using various solvents have demonstrated gastroprotective and antioxidant capabilities [8,9], thus garnering great attention in research on their extraction and application [10,11].

Flavonoids in plant matrices primarily as conjugated glycosides are formed through C-O glycosidic bonds between the hydroxyl groups of flavonoid aglycones and the saccharide moiety, possessing relative stability, high polarity and spatial heterogeneity, exhibiting different physicochemical properties and pharmacological activities [12,13]. Flavonoids extracted from natural products have been widely used in the field of medicine and nutrition due to their significant health benefits. Thus, obtaining these highly diverse flavonoids from various natural plants strongly depends on extraction techniques. Different approaches can affect the interaction between flavonoids and solvents under dissimilar conditions and lead to some structural changes or modifications such as degradation, deglycosylation or isomerization during the extraction process, which ultimately results in modulation of the bioactive properties of the extracted flavonoids [13,14]. In the previous study [9], we have found that MACF flavonoids exist in a glycosylated form, but the specific effects of glycosylation modifications on their biological activity have not been elucidated for further research to clarify the structure–activity relationship. Therefore, the chemical structure and biotransformation characteristics of the flavonoids during the extraction process need to be taken into account to ensure their desired biological activities.

For pharmaceutical and food applications, various techniques and extracting solvents have been developed for separation and purification of bioactive flavonoids from natural products [[13], [14], [15], [16], [17], [18]]. Microwave and ultrasonic assisted combined with aqueous two-phase extraction (ATPE) and deep eutectic solvent (DES) technologies have been utilized to enhance the extraction yield and increase the enrichment capacity [[15], [16], [17], [18], [19], [20], [21]]. A recent study has reported the optimization of ultrasonic-assisted aqueous two-phase extraction (UAATPE) by using response surface methodology to selectively separate and purify the flavonoid glycosides from MACF, which utilizes an aqueous two-phase system (ATPS) of ethanol/ammonium sulfate, and the flavonoid glycosides are extracted into the top phase while some impurities remain in the bottom phase [9]. Recently, some studies have shown that flavonoid glycosides often exhibit different pharmacological activities from their aglycones [[22], [23], [24]]. For example, rutin is a typical quercetin glycoside, but quercetin generally demonstrates stronger biological activities than rutin and has been applied to clinical use [18,22,23,25]. Also, Murota et al. reported that flavonoids glycosides cannot be absorbed directly after oral administration or exhibit low bioavailability; instead, they undergo hydrolysis into aglycones by gastric acid, which are partially absorbed in the small intestine or undergo further biotransformation by gut microbiota, thereby exerting the corresponding biological activities [26]. The bioactivity difference between flavonoid glycosides and their aglycones suggests that extraction and purification approaches should focus on their glycosylated and deglycosylated forms to further promote the development and application of flavonoids. In the previous study [9], although the UAATPE method can effectively extract and purify flavonoid glycosides from MACF, their aglycones can’t be directly obtained to evaluate their therapeutic potential. According to the conventional extraction methods, however, to obtain the flavonoid aglycones has to be subjected to at least three procedures including extracting the flavonoid glycosides from the sample, hydrolyzing of the flavonoid glycosides in the extract, and re-extracting and/or separating of the produced aglycones, which leads to more complex processing, time-expending and solvent-consuming [9,27,28]. Accordingly, based on the previous UAATPE study, a direct approach of obtaining the flavonoid aglycones from MACF was designed by constructing an acidified aqueous two-phase system (AATPS), which integrates extraction and hydrolysis of the flavonoid glycosides, and biphasic separation of the produced aglycones in a one-step procedure. In the acidified aqueous two-phase extraction (AATPE) process, the AATPS serves as both extractant and hydrolyzer for the flavonoid glycosides, and the produced flavonoid aglycones are transferred and enriched into the top phase by biphasic separation. With the assistance of an ultrasonic field, the combination of ultrasonic-assisted extraction (UAE) and AATPE is expected to intensify and accelerate the above chemical processes, achieving simplification of the complex steps and improvement of the extraction efficiency. Currently, ultrasonic bath and ultrasonic probe devices are commonly used in UAE application. Owing to gentle and uniform energy distribution, ultrasonic bath is suitable for parallel processing of multiple samples (such as small volume extraction), with compatibility with thermosensitive substances. Ultrasonic probe can provide high-intensity focused energy with precise amplitude and duration control, but the propensity for localized overheating and cross-contamination restrict the extraction of some flavonoids from plant matrices. By means of ultrasonic bath, UAE and AATPE is integrated to provide a simple, rapid and efficient alternative for direct extraction, separation and recovery of the flavonoid aglycones from MACF. To the best of our knowledge, there is no related report.

Pancreatic lipase (PL), being a target for anti-obesity drugs, holds a pivotal role in lipid metabolism. Accumulating researches on flavonoids has demonstrated strong inhibitory or synergistic inhibitory effects, suggesting a natural approach to regulate lipid metabolism and obesity [29,30]. This regulation mechanism, in turn, highlights the structure of flavonoids in lipid metabolism regulation, especially considering that the conversion of flavonoid glycosides to aglycones, which is an essential step in their physiological activities, as it enables the flavonoids to be absorbed and utilized effectively by the body. Therefore, it is imperative to obtain relevant evidence regarding the effect of structural changes before and after hydrolysis of flavonoids on the PL inhibitory activity, emphasizing the need for the preparation of flavonoid glycosides and aglycones. Herein, the flavonoids from MACF, as the pioneering compounds, were attempted to develop their PL inhibitory activity to make up for the lack of research on the structural changes.

**Fig. 1:**
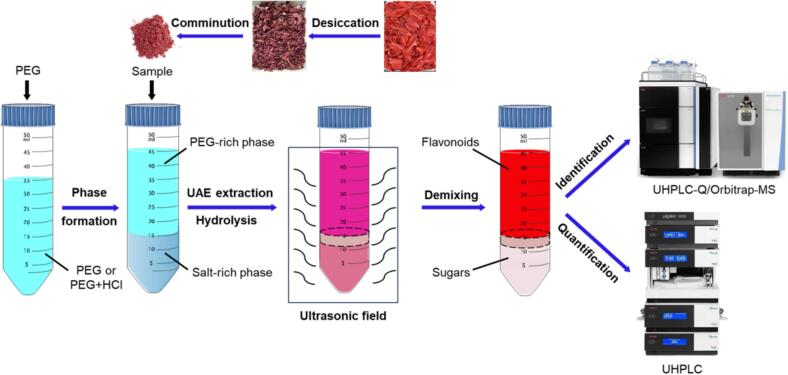
The process of extraction, hydrolysis, separation and analysis of the flavonoids from MACF by UAE-AATPE.

In this study, a polyethylene glycol (PEG)-based green extraction system was constructed to develop an efficient UAE-AATPE method for simultaneous extraction and hydrolysis of the flavonoid glycosides from MACF to obtain their aglycones. To this end, the multiple processes including solid–liquid extraction, acid hydrolysis and biphasic separation, were integrated and intensified by the assistance of an ultrasonic field with an appropriate AATPS as media. Accordingly, various AATPSs of PEGs were screened by adding the hydrochloric acid and ammonium sulfate for complete extraction and hydrolysis of the flavonoid glycosides from the sample to simultaneously extract and enrich the produced aglycones into the top phase. The UAE-AATPE process was further optimized by investigating the composition of phase-forming components, biphasic separation performance and extraction yields in order to more efficiently produce flavonoid aglycones. By means of ultrahigh-performance liquid chromatography with quadrupole orbitrap mass spectrometry (UHPLC-Q/Orbitrap-MS) and UHPLC-diode array detector (DAD), the obtained extracts were identified and quantified for comprehensive evaluation of PL inhibitory activity of MACF flavonoids. Finally, the inhibition on PL activity and the effect of structural transformation were explored by kinetic and molecular docking analyses. The experimental process of extraction, hydrolysis, separation and analysis of the flavonoids from MACF by UAE-AATPE is illustrated in Fig. 1.

## 2 Materials and methods

### 2.1 Materials and chemicals

*Malvaviscus arboreus *Cav. flowers were collected in the medicinal botanical garden of Guangdong Pharmaceutical University, Guangdong, China (No. 20221208). The samples were dried in hot air to constant weight, then ground into powder, passed through an 80-mesh screen, and stored in a desiccator at room temperature.

Catechin, cyanidin, pelargonidin, quercetin, kaempferol, and rutin (purities ≥ 98.0 %) were purchased from Shanghai Yuanye Bio-Technology Co., Ltd. (Shanghai, China). Pancreatic lipase (from porcine pancreas, Type II, ≥125 units/mg protein) was bought from Sigma-Aldrich Trading Co., Ltd. (Shanghai, China). Orlistat (purity ≥ 98.0 %) was obtained from China National Institute for the Control of Pharmaceutical and Biological Products (Beijing, China). Formic acid, methanol, and acetonitrile (LC-MS grade) were purchased from Merck Ltd. (Darmstadt, Germany). *p*-nitrophenyl palmitate (*p*-NPP), *p*-nitrophenol (*p*-NP), and dimethyl sulfoxide (DMSO) were purchased from Aladdin Biochemical Technology Co., Ltd. (Shanghai, China); Triton X-100, and sodium dodecyl sulfate (SDS) were purchased from Macklin Biochemical Technology Co., Ltd. (Shanghai, China). All other chemicals were of analytical grade or better and purchased from Guangzhou Chemical Reagent Factory (Guangzhou, China). Ultrapure water was obtained by Milli-Q integral water purification system (Millipore Co., Ltd, USA).

### 2.2 Instrumentation

KQ-200 VDE ultrasonic device (Kunshan Ultrasonic Instrument Co., Ltd., China); 2550 Ultraviolet–visible (UV–Vis) spectrophotometer (Shimadzu Co., Ltd., Japan); UltiMate 3000 ultra-high performance liquid chromatography and Vanquish Horizon UHPLC coupled with Orbitrap Exploris 120 quadrupole-orbitrap mass spectrometry and diode-array detector (DAD) (Thermo Fisher Scientific Co., Ltd., USA); Rotavapor R-110 rotary evaporator (Buchi Co., Ltd., Switzerland); ThermoFlex 900 recirculating chiller (Thermo Fisher Scientific Co., Ltd., USA); Multiskan Go microplate spectrophotometer (Thermo Fisher Scientific Co., Ltd., USA); Milli-Q Integral 3 water purification system (Merck Co., Ltd., Germany).

### 2.3 UAE-AATPE process

The AATPS was prepared by mixing PEG 600 with hydrochloric acid, followed by addition of ammonium sulfate to induce two-phase separation, and then the UAE-AATPE process was performed for extraction and hydrolysis of the flavonoid glycosides from MACF according to the following procedure. Briefly, 0.5 g of MACF powder was added to 45 mL of the AATPS in a vessel, mixed well, and then the mixture was placed in an ultrasonic bath at 70 % power (total power 220 W), 20 kHz, and 80 °C for 60 min to produce and separate the flavonoid aglycones from the sample. After cooling, the mixture was centrifuged at 4000 rpm for 5 min, and the filtrate was allowed to separate into two phases. Finally, the top phase solution was collected, diluted and filtered through a 0.22 μm filter membrane for the quantification analysis.

The partition coefficients (*K*), yield, and recovery of the flavonoid aglycones were calculated by the following equations:

(1) $$K=\frac{C_{T}}{C_{B}}$$

(2) $$\mathrm{Yield}\left(mg/g\right)=\frac{C_{T}V_{T}}{m_{s}}$$

(3) $$\mathrm{Recovery}\left(\%\right)=\frac{C_{T}V_{T}}{C_{T}V_{T}+C_{B}V_{B}}\times 100\%$$

where *C_T_* and *C_B_* represent the concentrations of the flavonoid aglycones in the top and bottom phases, respectively; *V_T_* and *V_B_* represent the volume of the top and bottom phases, respectively; *m_s_* is the mass of the sample powder (g).

### 2.4 UHPLC-Q-Orbitrap-MS identification of flavonoids

The flavonoids extracted from MACF were analyzed by UHPLC-Q/Orbitrap-MS and the separation was performed on a Poroshell 120 EC-C_18_ column (2.1 × 150 mm, 2.7 µm) using acetonitrile (A)-0.1 % formic acid solution (v/v) (B) as the mobile phase according to the following gradient elution procedure: 0–10 min, 95 % B; 10–20 min, 90 % B; 20–50 min, 85 % B; 50–70 min, 70 % B; 70–80 min, 60 % B; 80–85 min, 60 % B. For the hydrolyzed flavonoid aglycones: 0–5 min, 95 % B; 5–10 min, 80 % B; 10–15 min, 75 % B; 15–20 min, 65 % B; 20–45 min, 65 % B; 45–55 min, 50 % B; 55–60 min, 50 % B. The column temperature, the injection volume and the flow rate were set at 30 ℃, 1.0 µL and 0.3 mL/min, respectively. The eluted flavonoids were detected separately at 280 nm, 360 nm, and 510 nm.

Q-Orbitrap/MS identification of the flavonoids was conducted in positive and negative ion switching mode, and the scanning range was *m*/*z* 100.0–1000.0. The parameters are as follows: sheath gas flow rate 45 L/min, auxiliary gas flow rate 8 L/min, spray voltage 3.5 kV, ion transfer tube temperature 320 °C, RF-lens 70 %, vaporizer temperature 450 °C. The mass spectrometry mode was Full Mass-ddMS^2^ with the resolution of 60000, and the operation and acquisition of data were monitored by the Xcalibur workstation (Thermo Fisher Scientific, Germany).

### 2.5 UHPLC analysis

The flavonoids extracted from MACF were quantified on an UltiMate 3000 ultra-high performance liquid chromatograph equipped with DAD detector. Using acetonitrile (A)-0.1 % formic acid solution (v/v) (B) as the mobile phase, chromatographic separation was accomplished on an Agilent Eclipse XDB-C18 column (4.6 mm × 150 mm, 5 µm) by according to the gradient elution: 0–5 min 95 % B; 5–25 min, 90 % B; 25–45 min, 75 % B; 45–60 min, 60 % B. The column temperature, the flow rate and the injection volume were 30 ℃, 1.0 mL/min and 10 µL, respectively. The detection wavelengths were respectively at 280 nm, 360 nm, and 510 nm.

### 2.6 Evaluation of PL inhibitory activity of flavonoids

#### 2.6.1 PL inhibitory activity assay

The PL inhibition activity assay was carried out by *p*-NP spectrophotometry according to the previous study with some modifications [24,31,32]. Briefly, 100 µL of top phase extracts, 100 µL of Tris-HCl buffer (pH 8.0), and 100 µL of PL supernatant (10 mg/mL) were mixed together in a 2 mL centrifuge tube and then incubated at 37 °C for 15 min. Subsequently, 100 µL of pre-incubated *p*-NPP solution (6 mmol/L) was added to the above mixture to start the reaction and incubated at 37 °C for 15 min. Thereafter, 200 µL of Na_2_CO_3_ solution (0.2 mM) was added to terminate the reaction. Afterwards, absorbance of the mixture was measured at 405 nm. All the sample solutions were determined in triplicate, and the PL inhibition rate was calculated according to the following formula:

(4) $$Inhibition\;rate\left(\%\right)=\left[1-\frac{A_{\mathrm{sample}}-A_{\mathrm{control}}}{A_{\mathrm{blank}}}\right]\times 100$$

where *A_sample_* is the absorbance of the reaction mixture with the sample solution; *A_control_* is the absorbance of the reaction mixture with the Tris-HCl buffer replaced PL solution; *A_blank_* is the absorbance of the reaction mixture with the Tris-HCl buffer instead of the sample solution.

#### 2.6.2 Inhibition kinetic analysis

The enzyme inhibition kinetics of MACF flavonoids were carried out according to the following procedures. In brief, 100 µL of top phase extracts were mixed with 100 µL of Tris-buffer and 100 µL of enzyme supernatant (10 mg/mL) in a 2 mL centrifuge tube. The mixture was then incubated in a water bath at 37 °C for 15 min. Subsequently, 200 µL of pre-incubated *p*-NPP solution (1.0, 2.0, 4.0, 6.0 mM) was added and incubated at 37 °C for 15 min, and then 200 µL of Na_2_CO_3_ solution (0.2 mM) was added to terminate the reaction. Afterwards, the absorbances were measured at 405 nm on the microplate spectrophotometer, and all sample solutions were determined in triplicate. The kinetic parameters *Vmax* and *Km* of PL were obtained by nonlinear regression analysis of the enzymatic reaction rate curve according to the Michaelis-Menten equation, and then transformed into a Lineweaver-Burk equation model to determine the inhibition mechanism.

(5) $$\frac{1}{v}=\frac{K_{m}}{V_{\max}}\times \frac{1}{\left[S\right]}+\frac{1}{V_{\max}}$$

where *V*, *Vmax*, [*S*] and *Km* represent the enzymatic reaction rate, the maximum reaction rate, the substrate concentration, and kinetic constant, respectively.

#### 2.6.3 Molecular docking simulation

To elucidate the mechanism of interaction between the structure transformation of MACF flavonoids and PL, molecular docking simulations were conducted by using AutoDockTools (version 1.5.6). The three-dimensional structures of MACF flavonoids were retrieved from PubChem, while the crystal structure of PL (PDB ID: 1ETH) was obtained from the Protein Data Bank (PDB). Prior to docking, the 1ETH crystal structure was preprocessed by removing ligands, water molecules, and other chains, and adding nonpolar hydrogens and charges. Docking was then performed using AutoDock Vina (version 1.1.2), with MACF flavonoids (Pelargonidin-3,5-di-O-glucoside, kaempferol-3-O-sophoroside, catechin, cyanidin, pelargonidin, quercetin, and kaempferol) and PL treated as semi-flexible molecules within a Grid box. Three independent whole-protein crystal structure docking runs were conducted to predict potential binding modes of MACF flavonoids to PL and then the complex with the lowest binding energy was deemed as the most favorable interaction mode. Visualization and analysis of binding sites and interaction forces between amino acid residues of PL and MACF flavonoids were performed by using PyMOL (version 2.3.0), and the complex conformation with the best score was plotted by Discovery Studio (version 4.5.0).

### 2.7 Statistical analyses

To assess the significance of differences among variables, a one-way ANOVA conducted by SPSS 27.0 (International Business Machines Co. Ltd., USA) was utilized for statistical analyses, which were expressed as mean ± SD values. The statistically not significant, significant, and very significant levels were defined as *p* > 0.05 (#), *p* < 0.05 (*), and *p* < 0.01 (**), respectively.

## 3 Results and discussion

### 3.1 Optimization of UAE-AATPE process

#### 3.1.1 Screening of the PEG-based ATPS

**Fig. 2:**
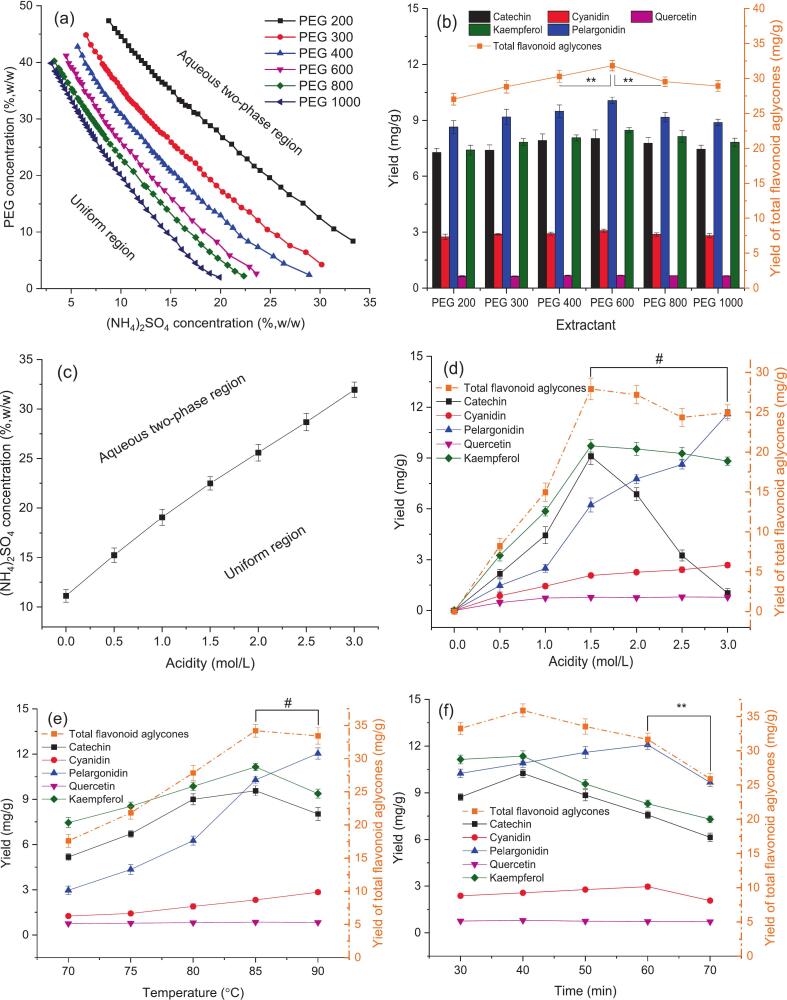
The phase diagram of the ATPS for PEG/ammonium sulfate (a); the extraction yield of the flavonoid aglycones in the top phase (b). The phase diagram of the AATPS for acidic PEG/ammonium sulfate (c) and the effects of acidity (d), hydrolysis temperature (e), and reaction time (f) on the extraction yield of the flavonoid aglycones from MACF by UAE-AATPE.

The ATPS formed by PEG-based/ammonium sulfate, emerges as an environmentally friendly extraction solvent [19]. In addition to the biocompatibility of ATPS, the carbon chain length of PEG alters the extraction performance and also affects phase distribution for the target compounds. According to the physicochemical properties of the target flavonoids, an ATPS can be tailored by screening the molecular weight of PEG to improve extraction efficiency [20]. Therefore, it is essential to choose a PEG with an appropriate molecular weight to construct a PEG-based ATPS for enhancing the extraction yield and ensuring recovery of flavonoids. Thus, the phase formation performance of PEG with a middle chain length and a different salt was preliminarily studied. Based on the ATPS formation of PEG 600 and different salts (Fig. S1), ammonium sulfate showed the best phase formation ability. Accordingly, the propensity for biphasic formation between PEG 200–1000 and ammonium sulfate was further examined through turbidimetric titration. As depicted in Fig. 2 (a), the binodal curves moved down and the aqueous two-phase region gradually widened with the increase of PEG molecular weight, meaning that an ATPS was easier to separate into two phases. Based on the phase diagram, a series of ATPSs of 30 % (w/w) PEG 200–1000 and 20 % (w/w) ammonium sulfate as phase-forming components were constructed for screening an ATPS with the higher extraction yield of flavonoids. According to our previous work, ultrasound field intensifying could induce cell rupture and enhance mass transfer, thereby achieving higher efficiency than conventional heating extraction and microwave-assisted extraction [9]. Therefore, ultrasonic-assisted aqueous two-phase extraction (UAATPE) was used to extract MACF flavonoid glycosides using the prepared ATPSs as extractants. Furthermore, the flavonoid glycosides enriched in the top phase were hydrolyzed to investigate the yields of the total and individual flavonoid aglycones by UV–vis spectrophotometry and UHPLC analysis, respectively.

As shown in Fig. 2 (b), the ATPS composed of PEG 600/ammonium sulfate exhibited the highest yield of total flavonoid aglycones, determined to be 31.82 ± 0.73 mg/g. Among them, the dominant flavonoid aglycones by UHPLC detection reached 30.33 ± 0.87 mg/g, including catechin, cyanidin, pelargonidin, quercetin, and kaempferol of 8.03 ± 0.45, 3.08 ± 0.08, 10.07 ± 0.18, 0.68 ± 0.02, and 8.47 ± 0.14 mg/g, respectively. These results demonstrated that the ATPS composed of PEG 600/ammonium sulfate could offer excellent extraction performance to obtain the flavonoid aglycones. However, the preparation of the flavonoid aglycones underwent two successive steps of extraction and hydrolysis. To streamline the process, the two steps were expected to combine into a single-step procedure for the preparation of the flavonoid aglycones, thus PEG 600/ammonium was used as the biphasic extractant for further constructing an AATPS to improve the UAE-AATPE process.

#### 3.1.2 Screening of the AATPS

In the biosynthesis of flavonoids, the glycosylation process significantly improves the solubility and stability of flavonoid glycosides. However, the glycosylation of flavonoids is often accompanied by a trade-off effect, as it tends to reduce their bioactivity, such as antioxidant and enzyme inhibitory properties [24]. In order to prepare the flavonoid aglycones efficiently, UAE-AATPE was designed for integrating the two complicated and time-consuming steps of extraction and hydrolysis of MACF flavonoids into a one-step procedure. Accordingly, the formation of an AATPS was further investigated by varying the acidity level with hydrochloric acid and corresponding addition of ammonium sulfate based on 30 % (w/w) of PEG 600.

As shown in Fig. 2 (c), as the acidity increased in the range of 0 ∼ 3 mol/L of HCl, more ammonium sulfate needed to be added to form the two phases. The reason is that increasing acidity would lead to production of HSO_4_^−^, reducing ion strength. As a result, the electrostatic repulsion between the salt and PEG 600 greatly weakened, which is extremely unfavorable for demixing of two phases separation, and required an increase in the salt content to supplement its deficiency. According to the phase diagram in Fig. 2 (c), therefore, a stable AATPS of PEG 600 and ammonium sulfate could be constructed and screened for more efficient extraction of the flavonoid aglycones from MACF, thus achieving simultaneous hydrolysis, extraction, separation and recovery of the flavonoid aglycones in order to evaluate the influence of structural transformation of MACF flavonoids on the PL inhibitory activity.

#### 3.1.3 The effects of main factors

For simultaneous hydrolysis, extraction, separation and recovery of MACF flavonoid aglycones, the effects of acidity, hydrolysis temperature, and reaction time in the UAE-AATPE process on the dynamic change of the yields of the flavonoid aglycones were investigated to ensure that the flavonoid glycosides were completely hydrolyzed into the corresponding aglycones during solid–liquid extraction, acid hydrolysis and biphasic separation. Firstly, the hydrolysis temperature was fixed at 80 ℃, and the reaction time was set to 30 min to investigate the influence of hydrolysis acidity on the yields of the flavonoid aglycones by UHPLC analysis. As shown in Fig. 2 (d), the yields of five flavonoid aglycones increased significantly with the increase of the acidity until 1.5 mol/L, the total yield peaked at 27.90 ± 1.36 mg/g. According to Fig. 2 (c), in the AATPS system with an acidity of 1.5 mol/L, the mass fractions of PEG 600, ammonium sulfate, and hydrochloric acid were 22.82 %, 22.50 %, and 10.93 % (w/w), respectively. As the acidity increased from 1.5 mol/L to 3.0 mol/L, the total yield of the flavonoid aglycones remained relatively stable, averaging at 27.15 ± 0.99 mg/g. Notably, the yield of catechin decreased significantly from 9.11 ± 0.49 mg/g to 1.03 ± 0.26 mg/g, presumably due to the absence of the conjugate structure of its C-ring, resulting in the photo-oxidation reaction [33]. In contrast, the yield of pelargonidin was improved along with increase of acidity, significantly contributing to the total yield of the flavonoid aglycones. Subsequently, the acidity was fixed at 1.5 mol/L to further investigate the effect of temperature on the hydrolysis of MACF flavonoids by controlling reaction time of 30 min. As shown in Fig. 2 (e), the total yield of the flavonoid aglycones reached 34.20 ± 0.97 mg/g at the hydrolysis temperature of 85 ℃, and catechin, quercetin, and kaempferol achieved the highest yields at 9.57 ± 0.33, 0.86 ± 0.05, and 11.15 ± 0.24 mg/g, respectively. However, both catechin and kaempferol suffered from considerable degradation at above 85 ℃, whereas anthocyanins were incompletely hydrolyzed. Consequently, the hydrolysis temperature of 85 ℃ was deemed an optimal level for the following investigation of other factors. Fig. 2 (f) illustrated how reaction time affected the yield of MACF flavonoid aglycones under the conditions of 1.5 mol/L of acidity and 85 °C of temperature. When the reaction time was set at 40 min, the total yield of the flavonoid aglycones peaked at 35.90 ± 0.96 mg/g, with the maximum yields for catechin and kaempferol being 10.25 ± 0.27 and 11.36 ± 0.33 mg/g, respectively. Due to their similar structures, both catechin and kaempferol exhibited similar trends, reaching their maximum yields within the same time window and degrading similarly with prolonged hydrolysis time. However, other flavonoid glycosides were not fully hydrolyzed at this time. As the hydrolysis duration reached 60 min, the highest yields of cyanidin and pelargonidin were obtained at 2.97 ± 0.15 and 12.08 ± 0.29 mg/g, respectively, while the total yield of the flavonoid aglycones decreased to 31.66 ± 0.93 mg/g, indicating that the flavonoid glycosides had been fully hydrolyzed. During the UAE-AATPE process, ultrasonic field assistance not only accelerated the release of flavonoid glycosides bound to the plant matrix, but also significantly promoted their subsequent acid hydrolysis, as well as accelerated the multiphase mass transfer from the solid phase to the liquid phase and from the bottom phase to the top phase. These synergistic effects demonstrated the indispensable role of the ultrasonic field in achieving both extraction efficiency and complete deglycosylation of the flavonoids, being beneficial for further large-scale application and activity evaluation studies.

In order to ensure complete hydrolysis of MACF flavonoid glycosides and avoid the influence of glycosylation on evaluation of the PL inhibition activity, therefore, the acidity of 1.5 mol/L, the hydrolysis temperature of 85 ℃, and the reaction time of 60 min were selected as the optimum conditions for complete deglycosylation of the flavonoid glycosides, especially for pelargonidin which is the most abundant flavonoid in MACF.

### 3.2 Identification and quantification of the flavonoids

#### 3.2.1 UHPLC-Q/Orbitrap-MS identification

**Fig. 3:**
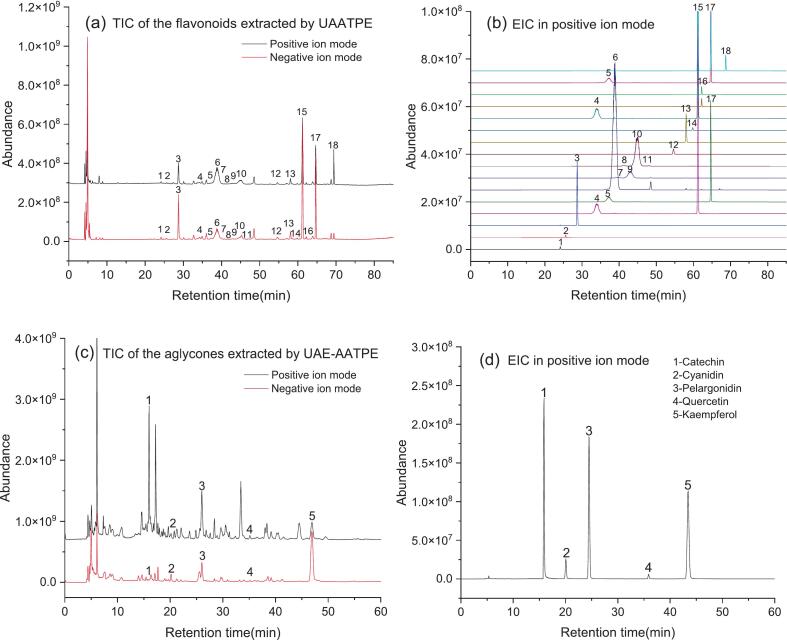
The total ion current (TIC) and the extracted ion current (EIC) chromatograms of MACF flavonoids extracted by UAATPE (a, b) and their aglycones extracted by UAE-AATPE (c, d).

By means of UHPLC-Q/Orbitrap-MS, the flavonoid aglycones enriched in the top phase by UAE-AATPE were identified by characterizing the mass spectrometric cleavage patterns. Meanwhile, the flavonoid glycosides in the top phase extracted by UAATPE were used as the control to investigate the structural transformation of MACF flavonoids. In general, MS/MS data demonstrated that the flavonoids had higher relative abundance in the negative ion mode [34], but the detection of catechins and anthocyanins in positive ion mode was more sensitive [24,35]. Thus, positive ion mode was selected to characterize the flavonoids in the top phase extracted by UAATPE and UAE-AATPE, and MS/MS data, the total ion current (TIC) and extracted ion current (EIC) are presented in Table S1 and Fig. 3, respectively.

**Fig. 4:**
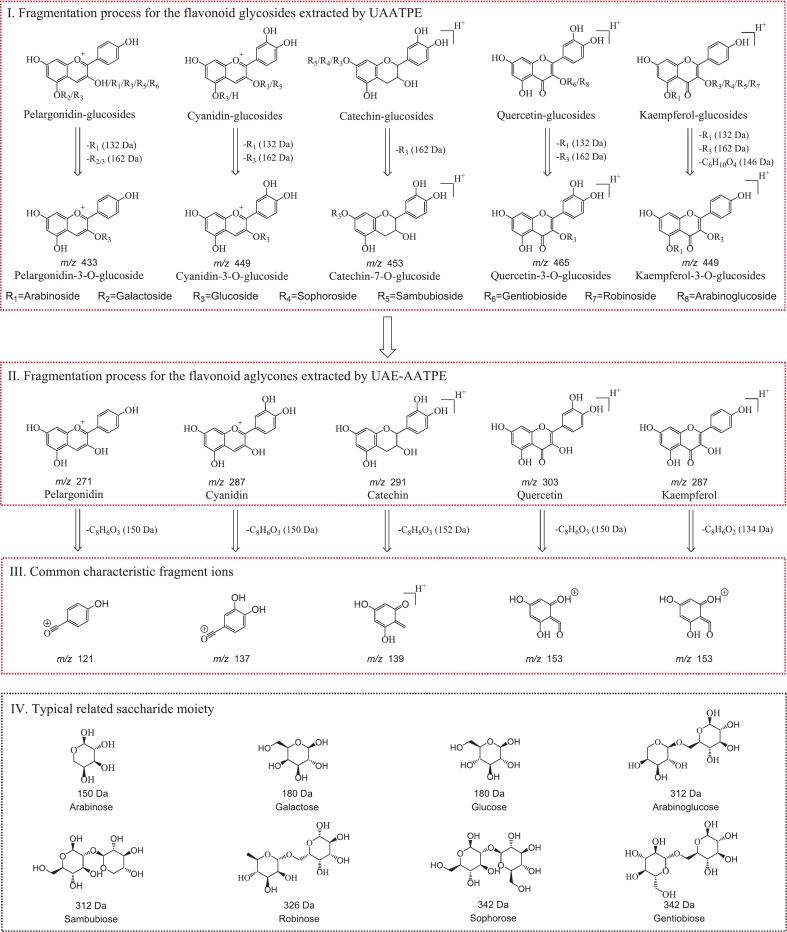
The characteristic MS/MS cleavage pathways of MACF flavonoids extracted by UAATPE and UAE-AATPE.

From Fig. 3 (a) and (b), 18 flavonoid glycosides in the top phase sample extracted by UAATPE were observed in the TIC and EIC chromatograms. As shown in Fig. 3 (c) and (d), only 5 peaks of the flavonoid aglycones in the sample extracted by UAE-AATPE were observed, and no flavonoid glycosides with corresponding *m*/*z* were detected after UAE-AATPE processing, suggesting that MACF flavonoid glycosides were completely hydrolyzed and enriched in the top phase. Based on MS/MS data, Fig. S2 included the fragmentation patterns for 5 kinds of flavonoids extracted from MACF, and then Fig. 4 illustrated the main cleavage process of the flavonoid glycosides and their aglycones extracted by UAATPE and UAE-AATPE, respectively. Ⅰ. The MS/MS fragment ions of flavonoid mono-glycosides with the *m*/*z* of 433.113, 449.108, 453.139, and 465.102 were observed by stepwise loss of saccharide moieties of the precursor ions of the flavonoid glycosides. Ⅱ. The further cleavage resulted in the production of characteristic flavonoid aglycones fragments with *m*/*z* of 271.060, 287.055, 291.086, and 303.049. Ⅲ. The C-ring of the flavonoids underwent heterocyclic fission and retro Diels-Alder reaction to further produce the common characteristic fragment ions with the *m*/*z* of 121.028, 137.023, 139.039, and 153.018. By comparison of MS/MS data, the obtained aglycones in the same way were subjected to Ⅱ and Ⅲ steps of MS/MS fragmentation, demonstrating that UAE-AATPE could completely deglycosylate MACF flavonoid glycosides [9,24,[34], [35], [36], [37], [38], [39]]. In addition, Fig. S3-S19 showed the EICs and MS/MS spectra of 18 flavonoid glycosides extracted by UAATPE and 5 flavonoid aglycones extracted by UAE-AATPE, deducing their detailed fragmentation patterns.

#### 3.2.2 UHPLC analysis

**Fig. 5:**
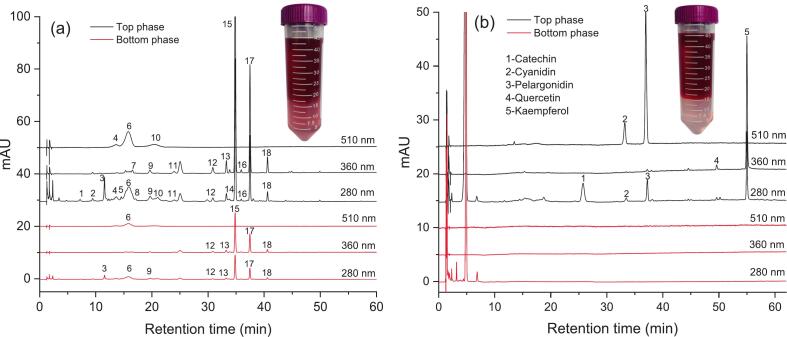
The UHPLC chromatograms of the flavonoid glycosides extracted by UAATPE (a) and their aglycones extracted by UAE-AATPE (b) from MACF.

In order to further evaluate the inhibitory activity, the flavonoids in UAATPE and UAE-AATPE extracts were chromatographically separated and detected by UHPLC-DAD according to the section 2.5 [See Fig. 5 (a) and (b)]. With the corresponding standards as the references, the quantification performance of 5 aglycones was validated by linearity, repeatability and stability tests, and the results were listed in Table 1. However, further quantification of the flavonoid glycosides linked with different saccharide groups poses great difficulty and challenge without the corresponding standards. Conventionally, the flavonoids extracted from plants are usually quantified by UV–Vis spectrophotometry or UHPLC analysis with rutin or quercetin as the standards, and then expressed as the total or relative content [40]. Faced with thousands of flavonoids, these methods are a compromise solution in the absence of corresponding standards [41]. According to the UHPLC-Q/Orbitrap-MS results, the main flavonoid aglycones extracted by UAE-AATPE were respectively catechin, cyanidin, pelargonidin, quercetin and kaempferol, while 18 flavonoid glycosides extracted by UAATPE were attributed to their derivatives. Based on UHPLC analysis of each flavonoid aglycone, thus its corresponding glycosides (*Y_ij_*) were quantified by calculating their individual proportion in its aglycone content or yield (*Y_ia_*) to more efficiently evaluate the content distribution and structural source of diverse flavonoids, i.e. the weighting coefficient (*f_ij_*) of various flavonoid glycosides was chromatographically determined by peak area normalization according to its classification, and then calculated its content by the content proportion of its aglycone as following equations.

(6) $$Y_{\mathrm{ij}}\left(mg/g\right)=f_{\mathrm{ij}}{\cdot Y}_{\mathrm{ig}},\;\;\;\;\;\;Y_{\mathrm{ig}}\left(mg/g\right)=f_{\mathrm{ij}}\frac{Y_{\mathrm{ia}}\bar{M_{\mathrm{ig}}}}{M_{i}}\;\;\;\;\;\;\;\;\;\;\;\;\;\;\;\;$$

$(\overset{¯}{M_{\mathrm{ig}}}=\sum_{j=1}^{n}f_{\mathrm{ij}}M_{\mathrm{ig}}$, $f_{\mathrm{ij}}=\frac{A_{\mathrm{ij}}}{\sum_{j=1}^{n}A_{\mathrm{ij}}}$, *i* = 1 ∼ 5 stands for a type of flavonoid aglycone)

**Table 1:** The regression equation, linear relationship, LOD, LOQ, repeatability, and stability for the flavonoid aglycones.

Compounds	Regression equation	Linear range (μg/mL)	R^2^	LOD (μg/mL)	LOQ (μg/mL)	Repeatability (n = 6, RSD %)	Stability (0–12 h, RSD %)
Catechin	A = 0.0985C + 0.0601	5.0–200.0	0.9999	1.001	3.337	0.85	1.85
Cyanidin	A = 0.0796C-0.0629	5.0–200.0	0.9998	0.273	0.910	0.61	1.57
Pelargonidin	A = 0.0919C + 0.0116	5.0–200.0	0.9998	0.174	0.580	0.72	1.41
Quercetin	A = 0.2847C −0.2730	5.0–200.0	0.9999	0.146	0.487	0.43	1.31
Kaempferol	A = 0.2283C-0.1485	5.0–200.0	0.9999	0.082	0.273	0.51	1.71

where *Y_ij_, f_ij_,* and *A_ij_* present the yield, the weighting coefficient and the peak area of each flavonoid glycoside for a type of flavonoid, respectively; *Y_ia_* and *Y_ig_* present the yield of a type of flavonoid aglycone and the total yield of its related glycosides, respectively. *M_i_*, *M_ig_* and ${\overset{¯}{M}}_{\mathrm{ig}}$ stand for the molecular weight of a type flavonoid aglycone, its related glycoside and the average molecular weight of all its related glycosides, respectively.

Table 2 listed the extraction yields and composition distribution of the flavonoid glycosides and their aglycones extracted from MACF by UAATPE and UAE-AATPE, respectively. The results demonstrated that the chemical composition of MACF flavonoids was dominated by pelargonidin, catechin and kaempferol, and 18 corresponding glycosides derived from them were successfully extracted by UAATPE. Accordingly, partition coefficient (*K*) for each flavonoid in varying forms revealed the extraction performance difference between UAATPE and UAE-AATPE, proving that the latter was more facilitated for enrichment and recovery of MACF flavonoids. Furthermore, UHPLC analysis could not only accurately determine the flavonoid aglycones, but also serve as a basis for quantification of their corresponding glycosides, particularly in the absence of flavonoid glycoside standards. This provided an efficient alternative for the quantification of flavonoids extracted from natural products.

**Table 2:** The distribution of the main flavonoid glycosides and their aglycones extracted by UAATPE and UAE-AATPE (n = 3)*.

Extraction method	Peak No.	Flavonoid compound	Top phase		Bottom phase		Partition coefficient (*K*)	Recovery (%)	RSD (%)
			Yield (mg/g)	RSD (%)	Yield (mg/g)	RSD (%)			
UAATPE	1	Catechin-7-O-sophoroside	1.547 ± 0.059	3.9	0.185 ± 0.004	2.1	8.38 ± 0.15	89.33 ± 0.18	1.1
	2	Catechin-7-O-sambubioside	1.493 ± 0.041	2.8	0.178 ± 0.007	4.0	8.41 ± 0.13	89.37 ± 0.14	1.2
	3	Catechin-7-O-glucoside	10.89 ± 0.312	2.9	1.420 ± 0.059	4.2	7.67 ± 0.10	88.47 ± 0.13	0.85
	4	Cyanidin-3,5-di-O-glucoside	5.323 ± 0.212	4.0	0.489 ± 0.028	5.8	10.89 ± 0.21	91.59 ± 0.15	0.92
	6	Pelargonidin-3,5-di-O-glucoside	18.24 ± 0.41	2.3	1.010 ± 0.013	1.3	9.03 ± 0.08	94.76 ± 0.05	1.1
	10	Pelargonidin-3-O-sambubioside	4.58 ± 0.014	0.3	0.233 ± 0.006	2.6	9.85 ± 0.26	95.17 ± 0.12	1.4
	12	Quercetin-3-O-gentiobioside	1.063 ± 0.041	3.7	0.046 ± 0.003	5.3	12.17 ± 0.21	96.05 ± 0.07	1.2
	13	Kaempferol-3-O-robinoside-7-O-glucoside	0.848 ± 0.018	2.2	0.038 ± 0.001	1.6	11.16 ± 0.09	95.71 ± 0.03	1.5
	14	Quercetin-3-O-arabinoglucoside	0.390 ± 0.018	4.6	0.013 ± 0.001	4.9	14.56 ± 0.08	96.68 ± 0.02	1.1
	15	Kaempferol-3-O-sophoroside	12.31 ± 0.26	2.1	0.577 ± 0.009	1.7	10.66 ± 0.07	95.52 ± 0.03	1.0
	16	Kaempferol-3-O-robinoside-7-O-arabinoside	0.214 ± 0.002	1.1	0.010 ± 0.001	1.3	11.09 ± 0.16	95.69 ± 0.06	1.4
	17	Kaempferol-3-O-sambubioside	5.023 ± 0.097	2.0	0.244 ± 0.005	2.1	10.28 ± 0.14	95.36 ± 0.06	1.0
	18	Kaempferol-3-O-glucoside	0.593 ± 0.008	1.3	0.032 ± 0.001	1.7	9.36 ± 0.05	94.93 ± 0.02	1.3
UAE-AATPE	1	Catechin	10.25 ± 0.244	2.4	ND	−	Large**	>99.999	−
	2	Cyanidin	2.971 ± 0.055	1.9	ND	−	Large**	>99.999	−
	3	Pelargonidin	12.08 ± 0.187	1.6	ND	−	Large**	>99.999	−
	4	Quercetin	0.810 ± 0.013	1.6	ND	−	Large**	>99.999	−
	5	Kaempferol	10.46 ± 0.183	1.8	ND	−	Large**	>99.999	−

* ND represents not detected; ** The extremely low content which is not detected in the bottom phase leads to the infinite values.

### 3.3 Exploration and characterization of UAE-AATPE process

**Fig. 6:**
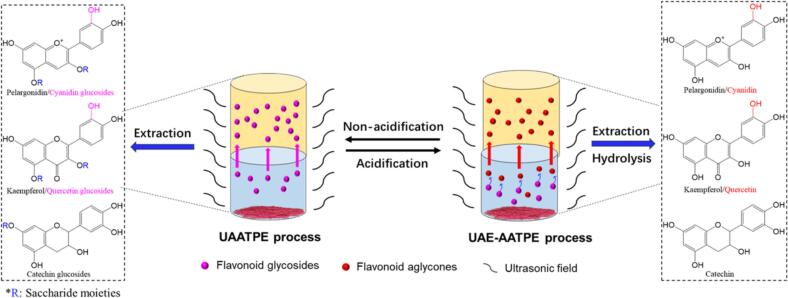
The mechanism of extraction and separation process of the glycosylated and deglycosylated flavonoids from MACF by UAATPE and UAE-AATPE, respectively.

According to the above results, the flavonoid aglycones in MACF could be directly obtained by UAE-AATPE, integrating multiple steps from extraction to hydrolysis and separation. These complex physicochemical changes were characterized by UHPLC monitoring of the flavonoid glycosides and their aglycones in both phases in order to further explore the mechanism of the UAE-AATPE process. Fig. 5 and Fig. 6 profiled the UAE-AATPE process for production and transformation from the flavonoid glycosides to their aglycones based on UAATPE processing.

As shown in Fig. 5 (a), with the unacidified ATPS as the extractant, about 18 flavonoid glycosides could be detected in the top phase of the extracted from MACF by UAATPE, but small amounts of the flavonoid glycosides were still retained in the bottom phase with pink. From Fig. 5 (b), there were only 5 flavonoid aglycones to be detected in the top phase at diverse retention times, but no flavonoid was found in the bottom phase with nearly colorless. This meant that the flavonoid glycosides were completely converted to the corresponding flavonoid aglycones. Indeed, this fact was readily observed and confirmed by the color indication of red pelargonins or pelargonidin due to relatively high content (as shown in Table 2). Obviously, the UAE-AATPE extract displayed a deeper red color in the top phase and almost no color in the bottom phase.

Fig. 6 illustrated the different mechanisms in UAATPE and UAE-AATPE processes. Even though the flavonoids in both UAATPE and UAE-AATPE processes were more preferentially extracted from MACF to the top phase, their different chemical forms would lead to diverse extraction ability while using the acidified and non-acidified systems (See Table 2). The results in Table 2 showed that the flavonoid aglycones extracted by UAE-AATPE had higher partition coefficients (*K*) and recoveries than the flavonoid glycosides extracted by UAATPE. Furthermore, Table 3 presented the characteristics of UAE-AATPE and the multiple-step conventional approach (UAATPE + UAAH). For obtainment of the flavonoid aglycones from MACF, UAATPE + UAAH was subjected to multiple steps including extraction, hydrolysis and separation of the flavonoid glycosides according to conventional procedures, whereas UAE-AATPE could directly extract the flavonoid aglycones from the sample by integrating these steps in a one-step procedure. By contrast, the results demonstrated that the ultrasonic field could not only intensify the multiple-phase extraction but also enhance the hydrolysis process. Crucially, it could also perfectly match with the AATPS to achieve multi-step integration and simplification, highlighting the significant advantages of UAE-AATPE. As a result, UAE-AATPE combining UAE with AATPE, condensed the multi-step process of the conventional approaches into a single, rapid and efficient protocol, simplifying the complex steps, improving the extraction yield and shortening the processing time. Furthermore, it exhibited the excellent superiority, easy scalability and simple practicality, which was more conducive to leveraging its unique advantages for the development and utilization of MACF flavonoid glycosides.

**Table 3:** Comparison of UAE-AATPE with UAATPE-UAAH for the extraction of the flavonoid aglycones from MACF.

Content	UAATPE + UAAH*	UAE-AATPE**
Procedure	Two steps: ① extraction; ② hydrolysis	One step integrating extraction, hydrolysis and separation
Extractant	ATPS of PEG 600/ammonium sulfate	AATPS of PEG 600/ammonium sulfate
Temperature	① Extraction at 80 °C; ② Hydrolysis at 80 °C	Extraction and hydrolysis at 80 °C
Acidity	2.4 mol/L HCl for hydrolysis	1.5 mol/L HCl for extraction and hydrolysis
Processing time	80 min: ① extraction 30 min; ② hydrolysis 50 min	60 min
Mode	Biphasic extraction + hydrolysis + re-extraction	Simultaneous extraction and hydrolysis
Yield	33.58 ± 0.53 mg/g	35.90 ± 0.96 mg/g
Scalability	Complex to scale up	Simple and easy to scale up

* UAATPE + UAAH: ultrasonic-assisted aqueous two-phase extraction (UAATPE) followed by ultrasonic-assisted acid hydrolysis (UAAH);

** UAE-AATPE: ultrasonic-assisted extraction coupled with acidified aqueous two-phase extraction.

### 3.4 Evaluation of the PL inhibitory activity of flavonoids

#### 3.4.1 The PL inhibitory effect and kinetics of flavonoids

**Fig. 7:**
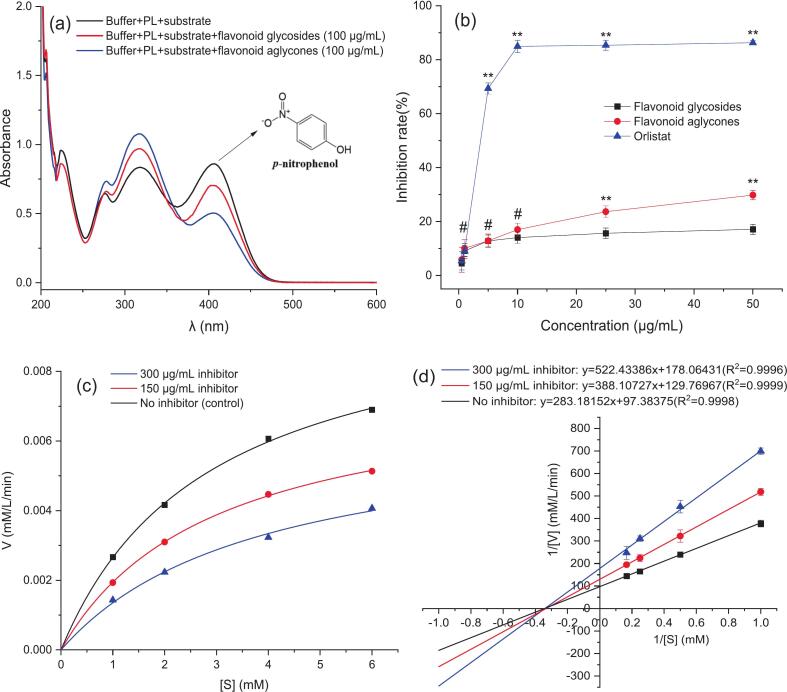
The UV–Vis spectra of PL inhibition f (a), the inhibitory effects on PL (b), and PL inhibition kinetics (c) and Lineweaver-Burk curves (d) for MACF flavonoid glucosides and their aglycones.

The structure–activity relationship and interaction mechanism between the structural changes of MACF flavonoids and their inhibitory activity on PL were studied by enzyme inhibition assay and kinetic analysis. Meanwhile, orlistat was used as the control drug to validate the inhibitory activity of the flavonoids. UV–Vis spectra in Fig. 7 (a) characterized the PL inhibition activity of MACF flavonoids before and after deglycosylation. The changes in absorption peaks at 318 nm and 405 nm were attributed to the formation of complexes between the flavonoids and the enzymes, reducing the enzyme activity and decreasing the production of *p*-NP. Fig. 7 (b) illustrated the PL inhibitory effect of MACF flavonoids, which indicated a concentration-dependent manner of the flavonoids and orlistat on the activity of PL within concentrations of 0.5–50.0 μg/mL. The dose–effect relationship revealed that there was no difference in the inhibition of PL by the flavonoid glycosides and aglycones at the concentrations below 10 μg/mL. However, as the concentration increased to over 10 μg/mL, the inhibition of PL by the flavonoid aglycones became significantly stronger than that of the flavonoid glycosides. The half-maximal inhibitory concentrations (IC_50_) for the flavonoid glycosides and their aglycones were 6318.19 ± 550.49 and 563.01 ± 280.63 μg/mL respectively, suggesting that the deglycosylation enhanced the PL inhibitory activity of MACF flavonoids.

The inhibition kinetics analysis in Fig. 7 (c) and (d) demonstrated that there was a negative correlation between the enzymatic reaction rate and the concentration of the inhibitor at the same substrate concentration. Accordingly, the double-reciprocal plot was constructed using the Lineweaver-Burk method and fitted to obtain the kinetic parameters (*K_m_* and *V_max_*) for the determination of the inhibition type of MACF flavonoid aglycones on PL. The slope of the line increased with the increase of inhibitor concentration, indicating the enhancement of inhibition. The *K_m_* (2.9 mM) was kept constant while the *V_max_* (*V_max_* = 0.01027, 0.00771, and 0.00560 mM/min at the concentration of 0.0, 150.0, 300.0 μg/mL, respectively) was decrease with the increase of the inhibitor concentration, and the fitting lines intersected at a point on the negative portion of the X axis, implying that the inhibitory effect of the flavonoid aglycones conformed to a noncompetitive mode of inhibition. In this case, the flavonoid aglycones were bound to the non-competitive domain rather than the active site and formed the inactive ternary “enzyme-substrate-inhibitor” complex [32,42]. The complex could cause a conformational change in the enzyme, preventing it from binding to the substrate and ultimately resulting in a decrease in PL activity.

#### 3.4.2 Molecular docking of the interaction with PL

**Fig. 8:**
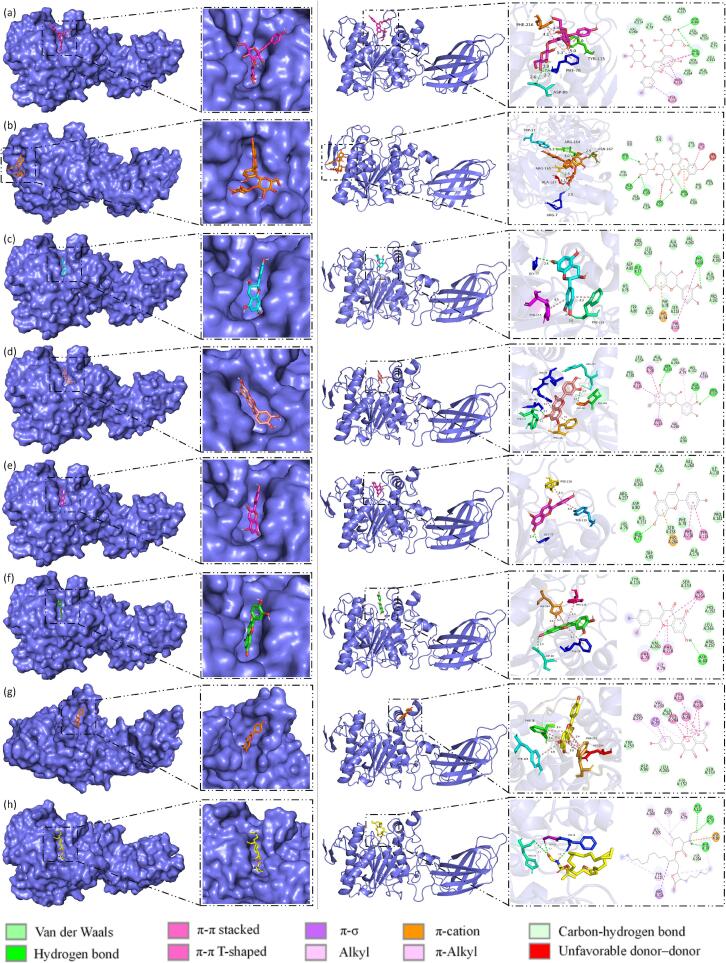
Molecular docking of MACF flavonoids and orlistat with PL: pelargonidin-3,5-di-O-glucoside (a); kaempferol-3-O-sophoroside (b); catechin (c); cyanidin (d); pelargonidin (e); quercetin (f); kaempferol (g); and orlistat (h).

Molecular docking analysis, as a mainstream research tool for studying ligand-receptor interactions, was employed to analyze the interaction between MACF flavonoids and PL. On the basis of the optimization of PL structure, two main flavonoid glycosides (pelargonidin-3,5-di-O-glucoside and kaempferol-3-O-sophoroside) and five flavonoid aglycones (catechin, cyanidin, pelargonidin, quercetin, and kaempferol) were selected as the ligands to simulate molecular recognition and the interaction between the flavonoids and PL. By means of molecular docking simulation, the effect of structural transformation of the MACF flavonoids on the affinity with PL could be further explored by observing the binding sites and dominant conformations to elucidate the interaction mechanism between MACF flavonoids and PL. Fig. 8 illustrated the entire protein docking with the flavonoids, including local magnification of its binding site, two-dimensional interaction, and intermolecular forces.

From Fig. 8, the binding sites of MACF flavonoids were located in the active pocket of the PL, i.e., the concave hydrophobic cavity. As shown in Table S2, the common interactions between these flavonoids and PL mainly included hydrogen bond interactions involving amino acids such as ASP, ALA, ARG and ASN, and π-cation and π-π stacking interactions involving amino acids such as PHE and TYR. However, deglycosylation of the flavonoids would strongly affect their interaction with PL. For example, compared to pelargonidin-3,5-di-O-glucoside, which formed hydrogen bonds with ASP-8 and PHE-78, the deglycosylated pelargonidin only formed hydrogen bonds with GLY-77. In addition, pelargonidin formed a π-cation interaction with HIS-264, suggesting that the presence of saccharide moieties may hinder this interaction, which is thought to be a crucial force in molecular recognition [43]. Therefore, the affinity of pelargonidin (−9.7 kcal/mol) was stronger than that of its glycosylated form (−8.4 kcal/mol). Another pair of the flavonoid glycosides and aglycones of interest are kaempferol-3-O-sophoroside and kaempferol, which could bind with other sites of PL. Specifically, the A and C rings of kaempferol-3-O-sophoroside could interact with PL through five hydrogen bonds (ARG-7, ALA-127, ARG-164, ARG-165, and ASN-167), π-π stacked (TRP-17), and π-cation (ARG-164) interactions. In contrast, kaempferol interacted with PL exhibited a different set of interactions including π-π stacked interactions (TYR-115, PHE-216, PHE-78, and HIS-264), π-Alkyl interactions (VAL-260 and ARG-257), and π-σ interactions (ILE-79), implying that kaempferol-3-O-sophoroside and kaempferol might have synergistic PL-inhibiting effects with other flavonoids similar to that of orlistat [44]. Both the flavonoids mentioned above demonstrated that the decrease in inhibitory effect was attributed to structural glycosylation that hindered their bonding with PL. Among MACF flavonoids, the order of the affinity on PL was pelargonidin>catechin>cyanidin>pelargonidin-3,5-di-O-glucoside>quercetin≈kaempferol>kaempferol-3-O-sophoroside, and pelargonidin exhibited the strongest affinity with the binding energy of −9.7 kcal/mol. Despite the stronger binding ability to PL, MACF flavonoids did not exhibit a higher PL inhibition rate. This might be due to their incomplete occupation of the key active sites of PL, conforming to the noncompetitive mode of the ternary “enzyme-substrate-inhibitor” complex. In contrast to the flavonoids, orlistat had the core part of β-lactone pharmacophore, its affinity with PL mainly involved alkyl or π-Alkyl interactions between the long alkyl chain and amino acid residues (VAL-260, ARG-257, ILE-79, LEU-265, TYR-115), which was significantly different from the flavonoids with the hydroxyl groups. The finding deserved insight into the targeting relationship between the active sites of PL and the functional groups of a potential inhibitor in order to further develop the pharmacological activity structurally. Therefore, the molecular docking simulations revealed that the flavonoid glycosides and aglycones had distinct binding patterns with PL, and proved that deglycosylation of MACF flavonoids could enhance the PL inhibitory activity, suggesting that their aglycones may have great potential for anti-obesity.

## 4 Conclusion

In this study, a novel approach for extraction and separation of the flavonoid aglycones from MACF was developed by UAE coupled to AATPE using an AATPS of PEG 600/ammonium sulfate. UAE-AATPE integrating extraction and hydrolysis in a one-step procedure could achieve direct production and recovery of the flavonoid aglycones from MACF sample. Under the optimized conditions, the flavonoid glycosides were completely hydrolyzed and spontaneously enriched in the top phase, attaining rapid recovery of their aglycones with a yield of 35.90 ± 0.96 mg/g. By means of UHPLC-Q/Orbitrap-MS and UHPLC-DAD, 18 MACF glycosylated flavonoids were derived respectively from pelargonidin, catechin, kaempferol, cyanidin, and quercetin in order of their content. The enzyme inhibition assay in vitro showed the stronger PL inhibitory activity of MACF flavonoid aglycones than that of their glycosylated flavonoids. Furthermore, inhibition kinetics and molecular docking analysis validated the characteristics of non-competitive inhibition of PL and inhibition mechanism, proving that deglycosylation of MACF flavonoids could promote PL inhibitory activity. By comparison of the conventional method, the ultrasonic field not only accelerated the release and hydrolysis of flavonoid glycosides from the plant matrix, but also promoted their multiphase mass transfer from the solid phase to the liquid phase and from the bottom phase to the top phase. As a result, the proposed UAE-AATPE demonstrated distinct advantages in simplification of complex steps and improvement of extraction efficiency, providing a simple, green and efficient alternative to production and recovery of the flavonoid aglycones from MACF for activity evaluation and further applications. Although PEG 600 has shown its potential as a green extractant, further reducing its viscosity impact will pose a challenge to scalability and applicability. Therefore, future research will focus on screening lower-viscosity acid AATPS alternatives (e.g. low-molecular-weight PEG derivatives or ionic liquid/acid salt combinations) to enhance large-scale application and activity evaluation study.

## CRediT authorship contribution statement

**Tiefeng Yuan:** Writing – review & editing. **Chen Lin:** Software, Methodology, Investigation, Funding acquisition, Formal analysis, Data curation. **Qingqing Yu:** Methodology, Investigation, Formal analysis, Data curation. **Xingyu Shi:** Validation, Investigation, Formal analysis, Data curation. **Peiyi Jin:** Visualization, Validation, Software, Investigation. **Jilong Huang:** Visualization, Validation, Investigation, Formal analysis. **Liping Wang:** Validation, Supervision, Resources, Project administration, Investigation, Funding acquisition, Conceptualization. **Huajun Fan:** Writing – review & editing, Supervision, Resources, Project administration, Methodology, Investigation, Formal analysis, Data curation, Conceptualization.

## Declaration of competing interest

The authors declare that they have no known competing financial interests or personal relationships that could have appeared to influence the work reported in this paper.

## Data Availability

Data will be made available on request.
