# Supplementary material for: Ultrasonic-assisted acidified aqueous two-phase extraction for one-step production and recovery of flavonoid aglycones from Malvaviscus arboreus Cav. flower: Process exploration, composition analysis and activity validation
Source: Ultrason Sonochem. 2025 Aug 31;121:107545. doi: 10.1016/j.ultsonch.2025.107545 (PMC12504977; doi:10.1016/j.ultsonch.2025.107545)
Supplement: Supplementary Data 1 [file mmc1.docx]

Supplementary materials for

**Ultrasonic-assisted acidified** **aqueous two-phase extraction** **for one-step production and recovery of flavonoid aglycones from *Malvaviscus arboreus* Cav. flower: Process exploration, composition analysis and activity validation**

Tiefeng Yuan^a,b,^*, Chen Lin^a^*, Qingqing Yu^b^, Xingyu Shi^b^, Peiyi Jin^b^, Jilong Huang^b^, Liping Wang^a^**, Huajun Fan^b^**

^a^ Guangdong Provincial Key Laboratory of Chemical Measurement and Emergency Test Technology,^，^Institute of Analysis, Guangdong Academy of Science (China national Analytical Center), Guangzhou, 510070, China

^b^ School of Pharmacy, Guangdong Pharmaceutical University, Guangzhou 510006, China

*These authors contribute equally.

**Corresponding author. Tel: +8602039352135; fax: +8602039352129; E-mail: [junhuafan@126.com](mailto:junhuafan@126.com); [wangjiang0916@126.com](mailto:wangjiang0916@126.com).

Supplementary materials included analysis of MS/MS fragmentation pattern, 2 tables and 19 figures.

*1. Analysis of MS/MS fragmentation pattern for flavonoid glycosides and flavonoid aglycones*

The identification of flavonoid aglycones is the crucial step in recognizing flavonoid glycosides, which was depended on the MS/MS fragment ion. As shown in Fig. 2 (e) and Fig. S3, on the basis of precursor ions and characteristic product ions, the flavonoid glycosides in MACF can be classified as flavonoid glycosides based on the complicated glycosylation (glucose, galactose, sophorose, etc.) of the C-3, 5, and 7 hydroxyl groups of flavonoid aglycones, such as catechin, cyanidin, pelargonidin, quercetin, and kaempferol, consistent with the major flavonoids found in previous study [1]. Due to the structural similarity of flavonoid aglycones and differences in the types and binding sites of glycosyl moieties, flavonoids in MACF form multiple pairs of isomers, including identical and different aglycone isomers, which poses a challenge to structural identification. For example, peaks 6 to 11 belong to homologous glucosides, which are highly stereospecific based on the glycosylation of pelargonidin with *m/z* of 595.166, 565.154, and 433.113. While, the other two pairs of isomers (peaks 4 & 15 and peaks 5 & 17) are due to the same *m/z* of the cyanidin and kaempferol and the attached glycosyl moieties. The distinction of isomers needs to be based on the fragmentation ions generated by further fragmentation of flavonoid aglycones. The flavonoids were tentatively identified based on the characteristic MS/MS fragment ions combined with their retention time, which are enumerated in Table S1.

The cleavage patterns of flavonoid glycosides and involves the relatively high abundance of common characteristic fragment ions generated by the C-ring cleavage of with *m/z* of 121.028, 137.023, 139.039, and 153.018, which can be summarized as characteristic fingerprints for the identification of flavonoid glycosides and aglycones [2,3]. In the top phase extracted by UAE-AATPE, the flavonoid glycosides were converted to the corresponding major aglycones (catechin, cyanidin, pelargonidin, quercetin, and kaempferol) by the hydrolysis-extraction process, which not only ulteriorly confirmed the identification results of flavonoid glycosides, but also provided more information about the MS/MS fragmentation of MACF flavonoids. As displayed in Fig. S16, the fragment ion with *m/z* 139.039 ([M+H-C_8_H_8_O_3_]^+^) was consistent with the fragment ion derived from catechin with *m*/*z* of 291.086 ([M+H]^+^) by means of retro Diels-Alder reaction. Subsequently, a loss of O (16 Da) occurred, which further generated fragment ions with *m/z* 123.044 ([M+H-C_8_H_8_O_4_]^+^). As shown in Fig. S17, cyanidin and kaempferol are isomers, exhibiting identical EIC with *m*/*z* of 287.055. As for cyanidin, two cleavage pathways were observed, resulting from the C-ring cleavage and enol to keto tautomerism between C-ring and B-ring of cyanidin. The fragment ion with *m/z* 137.022 ([M+H-C_8_H_6_O_3_]^+^) was generated by the degradation of 150 Da from the precursor ion, followed by further degradation to fragment ions of *m/z* 121.028 ([M+H-C_8_H_6_O_4_]^+^). In another cleavage pathway, the fragment ion with *m/z* 269.044 ([M+H-H_2_O]^+^) was obtained by means of losing a H_2_O (18 Da) at C-3 position of C-ring of cyanidin, which is the universal cleavage pattern of 3-hydroxyflavonol (pelargonidin, quercetin, and kaempferol, etc.). Subsequently, the B ring and C ring undergo heterocyclic fission and retro Diels-Alder reaction, which successionally lose two molecules of CO to obtain the fragment ions of *m/z* 241.050 ([M+H-CH_2_O_2_]^+^) and 213.052 ([M+H-C_2_H_2_O_3_]^+^). As for kaempferol, the fragment ions with *m/z* of 269.044 ([M+H-H_2_O]^+^), 241.050 ([M+H-CH_2_O_2_]^+^), and 213.054 ([M+H-C_2_H_2_O_3_]^+^) were obtained by dehydration and heterocyclic fission of kaempferol with *m*/*z* of 287.055 ([M+H]^+^). Alternatively, another two cleavage pathway are identical to quercetin, producing fragment ions with *m/z* 153.017 ([M+H-C_8_H_6_O_2_]^+^) and 121.028 ([M+H-C_8_H_6_O_4_]^+^). As depicted in Fig. S18, similar to the cleavage pathways of cyanidin, the fragment ions with *m/z* of 253.051 ([M+H-H_2_O]^+^), 197.059 ([M+H-C_2_H_2_O_3_]^+^), and 121.028 ([M+H-C_8_H_6_O_3_]^+^) were obtained by dehydration and retrocyclisation mechanism of pelargonidin with *m*/*z* of 271.060 ([M+H]^+^). Additionally, another cleavage pathway was observed in which the B ring and the C ring underwent intramolecular rearrangement simultaneously, resulting in the gradual loss of C_2_O_2_ and CO to produce the fragment ions with *m/z* 215.070 ([M+H-C_2_O_2_]^+^). As presented in Fig. S19, the fragment of *m*/*z* 285.039 ([M+H-H_2_O]^+^) was consistent with the fragment ion derived from quercetin by means of rearrangement of the B and C rings, losing a H_2_O (18 Da) from quercetin with *m/z* 303.049. Subsequently, the fragment ions with *m/z* 257.044 ([M+H-CH_2_O_2_]^+^) and 229.049 ([M+H-C_2_H_2_O_3_]^+^) were obtained by consecutively loss of two molecules of CO (28 Da). In another two pathway, the fragment ion of *m/z* 153.018 ([M+H-C_8_H_5_O_3_]^+^) and 137.023 ([M+H-C_8_H_6_O_4_]^+^) were produced by the loss of C_8_H_5_O_3_ (150 Da) and C_8_H_6_O_4_ (166 Da) by means of heterocyclic fission mechanism and retro Diels-Alder cleavage of B ring and C ring.

**References**

[1] T.F. Yuan, J.L. Huang, L. Gan, L.Z Chen, J.J. Zhong, Z.H. Liu, L.P. Wang, H.J. Fan, Ultrasonic enhancement of aqueous two-phase extraction and acid hydrolysis of flavonoids from *Malvaviscus arboreus* Cav. flower for evaluation of antioxidant activity. Antioxidants, 11 (10) (2022) 2039. <https://doi.org/10.3390/antiox11102039>.

[2] S.Q. Fang, Q.Y. Qu, Y.F. Zheng, H.H. Zhong, C.X. Shan, F. Wang, C.Y. Li, G.P. Peng, Structural characterization and identification of flavonoid aglycones in three Glycyrrhiza species by liquid chromatography with photodiode array detection and quadrupole time-of-flight mass spectrometry. Journal of Separation Science, 39 (11) (2016) 2068-78. <https://doi.org/10.1002/jssc.201600073>.

[3] T. Zhou, W. Guo, S.C. Ren, Y.M. Li, J.M. Wu, B. Yang, (2021). Flavonoid glycosides and other bioactive compounds in *citrus reticulate* ‘Chachi’ peel analysed by tandem mass spectrometry and their changes during storage. Carbohydrate Research, 510 (2021) 108462. <https://doi.org/10.1016/j.carres.2021.108462>.

*2. Supplementary tables*

Table S1 The results for UHPLC-Q/Orbitrap-MS analysis of flavonoid glycosides and their aglycones extracted by UAATPE and UAE-AATPE.

Table S2 The molecular docking results of MACF flavonoids and orlistat on PL.

Table S1 The results for UHPLC-Q/Orbitrap-MS analysis of flavonoid glycosides and their aglycones extracted by UAATPE and UAE-AATPE.

| Sample | No. | t_R_  (min) | Molecular formula | Calculated  [M + H]^+^ | Determined  [M + H]^+^ | Error  (ppm) | `Compound | MS/MS fragments |
| --- | --- | --- | --- | --- | --- | --- | --- | --- |
| Flavonoid glycosides | 1 | 24.14 | C_27_H_34_O_16_ | 615.19193 | 615.19174 | -0.3088 | Catechin-7-O-sophoroside | 453.13909, 291.08618, 139.03896, 123.04391 |
|  | 2 | 25.61 | C_26_H_32_O_15_ | 585.18140 | 585.18092 | -0.8203 | Catechin-7-O-sambubioside | 291.08638, 139.03883, 123.04361 |
|  | 3 | 28.68 | C_21_H_24_O_11_ | 453.13914 | 453.13965 | 1.1255 | Catechin-7-O-glucoside | 291.08633, 139.03867, 123.04349 |
|  | 4 | 33.94 | C_27_H_31_O_16_ | 611.16066 | 611.16087 | 0.3436 | Cyanidin-3,5-di-O-glucoside | 449.10382, 287.05309, 137.02324 |
|  | 5 | 37.33 | C_26_H_29_O_15_ | 581.15010 | 581.15086 | 1.3078 | Cyanidin-3-O-sambubioside | 449.10358, 287.05472, 137.02291 |
|  | 6 | 38.88 | C_27_H_31_O_15_ | 595.16575 | 595.16673 | 1.6466 | Pelargonidin-3,5-di-O-glucoside | 433.11373, 271.05945, 121.02841 |
|  | 7 | 39.48 | C_21_H_21_O_10_ | 433.11292 | 433.11306 | 0.3232 | Pelargonidin-5-O-galactoside | 271.05928, 121.02841 |
|  | 8 | 41.32 | C_26_H_29_O_14_ | 565.15518 | 565.15460 | -1.0263 | Pelargonidin-3-O-arabinoside-5-O-glucoside | 271.05916, 121.02851 |
|  | 9 | 42.91 | C_21_H_21_O_10_ | 433.11292 | 433.11273 | -0.4387 | Pelargonidin-3-O-glucoside | 271.05909, 121.02830 |
|  | 10 | 45.01 | C_26_H_29_O_14_ | 565.15518 | 565.15460 | -1.0263 | Pelargonidin-3-O-sambubioside | 271.05940, 121.02846 |
|  | 11 | 48.47 | C_27_H_31_O_15_ | 595.16575 | 595.16602 | 0.4537 | Pelargonidin-3-O-gentiobioside | 433.11332, 271.05932, 121.02837 |
|  | 12 | 54.64 | C_27_H_30_O_17_ | 627.15558 | 627.15584 | 0.4146 | Quercetin-3-O-gentiobioside | 465.10279, 303.04937, 153.01757 |
|  | 13 | 58.01 | C_33_H_40_O_20_ | 757.21857 | 757.21899 | 0.5547 | Kaempferol-3-O-robinoside-7-O-glucoside | 595.16561, 449.10803, 287.05544, 153.01761 |
|  | 14 | 59.82 | C_26_H_28_O_16_ | 597.14501 | 597.14552 | 0.8541 | Quercetin-3-O-arabinoglucoside | 465.10262, 303.05045, 153.01966 |
|  | 15 | 61.15 | C_27_H_30_O_16_ | 611.16066 | 611.15984 | -1.3417 | Kaempferol-3-O-sophoroside | 449.10873, 287.05505, 153.01685 |
|  | 16 | 62.20 | C_32_H_38_O_19_ | 727.20801 | 727.20840 | 0.5363 | Kaempferol-3-O-robinoside-7-O-arabinoside | 595.16521, 449.10823, 287.05543, 153.01702 |
|  | 17 | 65.64 | C_26_H_28_O_15_ | 581.15010 | 581.14999 | -0.1893 | Kaempferol-3-O-sambubioside | 449.10868, 287.05588, 153.01743 |
|  | 18 | 68.68 | C_21_H_20_O_11_ | 449.10784 | 449.10817 | 0.7348 | Kaempferol-3-O-glucoside | 287.05523, 153.01745, 153.01745 |
| Aglycone | 1 | 13.25 | C_15_H_14_O_6_ | 291.08631 | 291.08646 | 0.5153 | Catechin* | 139.03902, 123.04411 |
|  | 2 | 20.08 | C_15_H_11_O_6_ | 287.05501 | 287.05513 | 0.4180 | Cyanidin* | 269.04454, 241.05021, 213.05295, 137.02267, 121.02828 |
|  | 3 | 24.46 | C_15_H_11_O_5_ | 271.06010 | 271.06018 | 0.2951 | Pelargonidin* | 253.05110, 215.07034, 197.05980, 121.02866 |
|  | 4 | 35.84 | C_15_H_10_O_7_ | 303.04993 | 303.04977 | -0.5280 | Quercetin* | 285.03958, 257.04449, 229.04939, 153.01808, 137.02318, 121.02843 |
|  | 5 | 43.47 | C_15_H_10_O_6_ | 287.05501 | 287.05505 | 0.1393 | Kaempferol* | 269.04462, 241.05041, 213.05447, 153.01747, 121.02865 |

* Indicates that the ingredient has been compared with the reference substance.

Table S2 The molecular docking results of MACF flavonoids and orlistat on PL.

| Compounds | Affinity (kcal/mol) | Major interacting amino acid residues |
| --- | --- | --- |
| Pg-3,5-di-O-glu* | -8.4 | Hydrogen bond: ASP-80, PHE-78; π-π stacked: PHE-216, TYR-115; Van der waals: VAL-260, LEU-214, ILE-79, ALA-261, ARG-257, LEU-265, HIS-152, GLY-77, LEU-154, SER-153, ALA-179, HIS-264. |
| Kp-3-O-sop* | -7.7 | Hydrogen bond: ARG-7, ALA-127, ARG-164, ARG-165, ASN-167; π-π stacked: TRP-17; π-cation: ARG-164; Carbon-hydrogen bond: LEU-8; Van der waals: TYR-131, ALA-130, GLU-134, GLU-161, PHE-11, ARG-123, CYS-10, GLY-9. |
| Catechin | -9.3 | Hydrogen bond: GLY-77, PHE-216; π-π stacked: TYR-115; π-cation: HIS-264; Van der waals: ARG-257, LEU-265, ALA-261, VAL-260, GLU-180, ALA-179, PRO-181, SER-153, PHE-78, HIS-152, TRP-86, HIS-76, ASP-80. |
| Cyanidin | -8.8 | Hydrogen bond: SER-153, ALA-261, ARG-257; π-π stacked: PHE-78, TYR-115, PHE-216; π-Alkyl: ILE-79, IEU-265, VAL-260; Van der waals: PRO-181, LEU-154, ALA-179, HIS-264, HIS-152, ASP-80. |
| Pelargonidin | -9.7 | Hydrogen bond: GLY-77; π-π stacked: PHE-216, TYR-115; π-cation: HIS-264; Van der waals: ALA-261, VAL-260, ILE-210, PRO-181, ALA-179, PHE-78, SER-153, TRP-86, HIS-76, HIS-152, ARG-257, ASP-80, LEU-265. |
| Quercetin | -7.8 | Hydrogen bond: ASP-80; π-π stacked: PHE-78, PHE-216, HIS-264; π-Alkyl: ILE-79; Van der waals: TYR-115, SER-153, HIS-152, LEU-265, ARG-257, VAL-260. |
| Kaempferol | -7.8 | π-π stacked: TYR-115, PHE-216, PHE-78, HIS-264; π-Alkyl: VAL-260, ARG-257; π-σ: ILE-79. |
| Orlistat | -7.2 | Hydrogen bond: HIS-152, GLY-77, PHE-78; π-π stacked: PHE-216; π-cation: ASP-80; Alkyl or π-Alkyl: ARG-257, ILE79, LEU265, TYR-115, VAL-260; Van der waals: HIS-264. |

*Pg-3,5-di-O-glu: Pelargonidin-3,5-di-O-glucoside; *Kp-3-O-sop: Kaempferol-3-O-sophoroside

*3. Supplementary figures*

Fig. S1. 35 mL ATPS with 30% PEG 600 and 20% different salts (a-e: sodium chloride, potassium dihydrogen phosphate, dipotassium phosphate, sodium dihydrogen phosphate and ammonium sulfate).

Fig. S2. Mass spectrometric fingerprints of characteristic flavonoid glycosides [catechin glycosides (a), cyanidin glycosides (b), pelargonidin glycosides (c), quercetin glycosides (d), and kaempferol glycosides (e)] of MACF.

Fig. S3. The EIC (a), MS/MS spectra (b), and the proposed fragmentation pattern (c) of catechin-7-O-sophoroside.

Fig. S4. The EIC (a), MS/MS spectra (b), and the proposed fragmentation pattern (c) of catechin-7-O-sambubioside.

Fig. S5. The EIC (a), MS/MS spectra (b), and the proposed fragmentation pattern (c) of catechin-7-O-glucoside.

Fig. S6. The EIC (a), MS/MS spectra (b, c), and proposed fragmentation patterns (d, e) of cyanidin-3,5-di-O-glucoside and kaempferol-3-O-sophoroside.

Fig. S7. The EIC (a), MS/MS spectra (b, c), and proposed fragmentation patterns (d, e) of cyanidin-3-O-sambubioside and kaempferol-3-O-sambubioside.

Fig. S8. The EIC (a), MS/MS spectra (b, c), and proposed fragmentation patterns (d, e) of pelargonidin-3,5-di-O-glucoside and pelargonidin-3-O-gentiobioside.

Fig. S9. The EIC (a), MS/MS spectra (b, c), and proposed fragmentation patterns (d) of pelargonidin-5-O-galactoside and pelargonidin-3-O-glucoside.

Fig. S10. The EIC (a), MS/MS spectra (b, c), and proposed fragmentation patterns (d) of pelargonidin-3-O-arabinoside-5-O-glucoside and pelargonidin-3-O-sambubioside.

Fig. S11. The EIC (a), MS/MS spectra (b), and the proposed fragmentation pattern (c) of quercetin-3-O-gentiobioside.

Fig. S12. The EIC (a), MS/MS spectra (b), and the proposed fragmentation pattern (c) of kaempferol-3-O-robinoside-7-O-glucoside.

Fig. S13. The EIC (a), MS/MS spectra (b), and the proposed fragmentation pattern (c) of quercetin-3-O-arabinoglucoside.

Fig. S14. The EIC (a), MS/MS spectra (b), and the proposed fragmentation pattern (c) of kaempferol-3-O-robinoside-7-O-arabinoside.

Fig. S15. The EIC (a), MS/MS spectra (b, c, d), and the proposed fragmentation pattern (e) of kaempferol-3-O-sophoroside, kaempferol-3-O-sambubioside, and kaempferol-3-O-glucoside.

Fig. S16. The EIC (a), MS/MS spectra (b), and the proposed fragmentation pattern (c) of catechin.

Fig. S17. The EIC (a), MS/MS spectra (b, c), and proposed fragmentation patterns (d, e) of cyanidin and kaempferol.

Fig. S18. The EIC (a), MS/MS spectra (b), and the proposed fragmentation pattern (c) of pelargonidin.

Fig. S19. The EIC (a), MS/MS spectra (b), and the proposed fragmentation pattern (c) of quercetin.


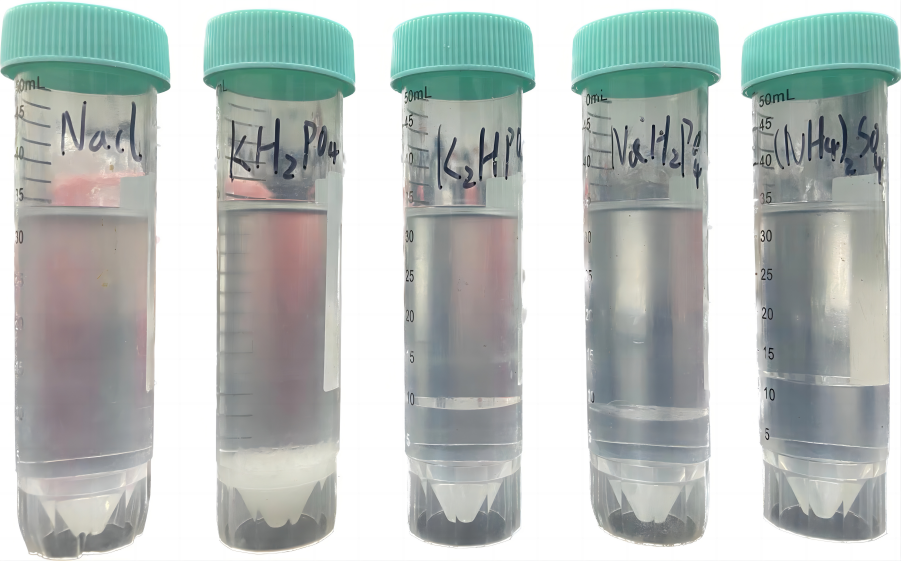


a b c d e

Fig. S1. 35 mL ATPS with 30% PEG 600 and 20% different salts (a-e: sodium chloride, potassium dihydrogen phosphate, dipotassium phosphate, sodium dihydrogen phosphate and ammonium sulfate).

Fig. S2. Mass spectrometric fingerprints of characteristic flavonoid glycosides [catechin glycosides (a), cyanidin glycosides (b), pelargonidin glycosides (c), quercetin glycosides (d), and kaempferol glycosides (e)] of MACF.

Fig. S3. The EIC (a), MS/MS spectra (b), and the proposed fragmentation pattern (c) of catechin-7-O-sophoroside.

Fig. S4. The EIC (a), MS/MS spectra (b), and the proposed fragmentation pattern (c) of catechin-7-O-sambubioside.

Fig. S5. The EIC (a), MS/MS spectra (b), and the proposed fragmentation pattern (c) of catechin-7-O-glucoside.

Fig. S6. The EIC (a), MS/MS spectra (b, c), and proposed fragmentation patterns (d, e) of cyanidin-3,5-di-O-glucoside and kaempferol-3-O-sophoroside.

Fig. S7. The EIC (a), MS/MS spectra (b, c), and proposed fragmentation patterns (d, e) of cyanidin-3-O-sambubioside and kaempferol-3-O-sambubioside.

Fig. S8. The EIC (a), MS/MS spectra (b, c), and proposed fragmentation patterns (d, e) of pelargonidin-3,5-di-O-glucoside and pelargonidin-3-O-gentiobioside.

Fig. S9. The EIC (a), MS/MS spectra (b, c), and proposed fragmentation patterns (d) of pelargonidin-5-O-galactoside and pelargonidin-3-O-glucoside.

Fig. S10. The EIC (a), MS/MS spectra (b, c), and proposed fragmentation patterns (d) of pelargonidin-3-O-arabinoside-5-O-glucoside and pelargonidin-3-O-sambubioside.

Fig. S11. The EIC (a), MS/MS spectra (b), and the proposed fragmentation pattern (c) of quercetin-3-O-gentiobioside.

Fig. S12. The EIC (a), MS/MS spectra (b), and the proposed fragmentation pattern (c) of kaempferol-3-O-robinoside-7-O-glucoside.

Fig. S13. The EIC (a), MS/MS spectra (b), and the proposed fragmentation pattern (c) of quercetin-3-O-arabinoglucoside.

Fig. S14. The EIC (a), MS/MS spectra (b), and the proposed fragmentation pattern (c) of kaempferol-3-O-robinoside-7-O-arabinoside.

Fig. S15. The EIC (a), MS/MS spectra (b, c, d), and the proposed fragmentation pattern (e) of kaempferol-3-O-sophoroside, kaempferol-3-O-sambubioside, and kaempferol-3-O-glucoside.

Fig. S16. The EIC (a), MS/MS spectra (b), and the proposed fragmentation pattern (c) of catechin.

Fig. S17. The EIC (a), MS/MS spectra (b, c), and proposed fragmentation patterns (d, e) of cyanidin and kaempferol.

Fig. S18. The EIC (a), MS/MS spectra (b), and the proposed fragmentation pattern (c) of pelargonidin.

Fig. S19. The EIC (a), MS/MS spectra (b), and the proposed fragmentation pattern (c) of quercetin.
